# The varying roles of the dimensions of affluence in air pollution: a regional STIRPAT analysis for Germany

**DOI:** 10.1007/s11356-022-23519-2

**Published:** 2022-10-14

**Authors:** Johannes Lohwasser, Axel Schaffer

**Affiliations:** Bundeswehr University Munich, Werner-Heisenberg-Weg 39, 85577 Neubiberg, Germany

**Keywords:** STIRPAT model, NO_x_ emissions, Affluence, Car ownership, Number of houses, Regional, Long-run elasticities

## Abstract

STIRPAT models investigate the impacts of population, affluence, and technology on the environment, with most STIRPAT studies revealing positive impacts of both population and affluence. Affluence is commonly defined as GDP per capita, but investigations of its impact largely neglect the possibility that increasing prosperity affects the environment in varying—even opposing—ways. This study addresses this gap by decomposing affluence into three dimensions—income per taxpayer, private car ownership, and the share of single-family houses—and analyzing their roles in the production of local NO_x_ emissions. Results for 367 German districts and autonomous cities between 1990 and 2020 indicate that, while private car ownership and single-family houses per capita can be considered drivers of local pollutants, such is not the case for income per taxpayer, which we find has a negative impact on NO_x_ emissions. The empirical findings suggest that policies should strengthen integrated mobility concepts and establish incentives that favor investment in modern heating or self-sufficiency systems.

## Introduction

Despite recent improvements in air quality, about 90% of the European Union’s (EU) urban population are exposed to concentration levels above the World Health Organization’s (WHO) latest annual guidelines for fine particulate matter (PM_2.5_), ozone (O_3_), and nitrogen dioxide (NO_x_) (European Environment Agency 2022). Therefore, air pollution is still a considerable threat to ecosystems and human health in the EU. In response, the EU clean air policy set ambitious reduction commitments for main air pollutants that member states are required to integrate in their national environmental policies.

**Fig. 1:**
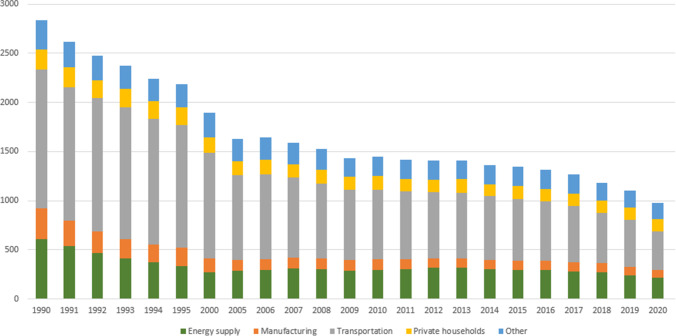
Sources of NO_x_ emissions in Germany (1990–2020, in thousand tons). Source: based on Umweltbundesamt (2022)

As one of the EU’s main nitrogen oxide polluters, Germany is committed to reducing NO_x_ emissions by 65% by 2030 compared to that of 2005 (Umweltbundesamt 2019).^1^ Therefore, German law- and policy-makers are interested in learning more about the main sources of NO_x_ emissions at the sectoral level and about its socioeconomic drivers at the macro level. These emissions’ sources are mainly the transportation, energy use, private households, and manufacturing sectors (Fig. 1).

As for NO_x_ emissions’ socioeconomic drivers at the macro level, the relationships of economic activities and population with environmental impacts (e.g., greenhouse gases, air pollution) are often analyzed using the environmental Kuznets curve (EKC) to measure the non-linear impact of the economy or population on the environment or the STIRPAT model to measure the stochastic impacts on the environment by regressing population, affluence, and technology. More recently, some studies also incorporate the EKC effect into STIRPAT modelling by adding non-linear effects of gross domestic product (GDP) or population size into the STIRPAT equation (e.g Cole and Neumayer 2004; Ge et al. 2018; Arshed et al. 2021).

Most empirical findings generally confirm the now well-established positive impact of population and affluence on the environment in the STIRPAT framework (e.g., Liddle and Lung 2010; Andrés and Padilla 2018). However, a close look at the large variety of empirical studies reveals that it is not the population as a whole that increases environmental pressures but certain groups in the population. Therefore, many authors differentiate population by region (global north vs. global south), by economic status (rich vs. poor; economically active vs. inactive), by settlement structure and density (urban vs. regional), by age group (young, middle, old), or educational achievement.

In contrast, affluence is almost exclusively defined as GDP per capita, which neglects the possibility that increasing prosperity affects the environment in different—even opposing—ways. Notable exceptions to this oversight include studies that disaggregate GDP by sector (Arshed et al. 2021; Wang et al. 2021), account for infrastructure capital per capita (Li et al. 2017), or expand the model using household size (Yousaf et al. 2021) or elements of consumer behavior, such as consumption of material goods (Kilbourne and Thyroff 2020).

Against this background, the present study analyzes affluence in a differentiated way. This approach is in line with recent empirical findings on poverty and wealth (e.g., Peichl and Pestel, 2013; Törmälehto 2017), which suggest that more differentiated measures than GDP per capita are needed to capture all aspects of affluence (e.g., living conditions, social exclusion, and mobility) and take into account that STIRPAT analysis originally differentiated affluence between national income and consumption patterns (Dietz and Rosa 1994).

In taking this approach, we seek to identify the impacts on local air pollution (measured by NO_x_ emissions) of regional population and the three aspects of affluence in German districts and autonomous cities between 1990 and 2020. We decompose affluence into (taxable) income per taxpayer, private car ownership, and the share of single-family houses per capita.

While our results confirm the long established positive relationship between NO_x_ emissions and population, the role of affluence is less conclusive. While the level of car ownership and the share of single-family houses per capita both have strong positive impacts on emissions, taxable income per taxpayer reveals a negative relationship between local NO_x_ emissions and taxable income per taxpayer (when we control for car ownership and the share of single-family houses per capita).

The remainder of the paper is organized as follows. The “Literature Review” section provides an overview of related literature, focusing on empirical findings and the treatment of affluence. The “Decomposition of affluence” section describes the decomposition of affluence we used. The “Theoretical model and empirical application” section introduces the STIRPAT model and describes the data and the empirical application of the model. The “Discussion of results” section follows with a discussion of the results, and the “Concluding remarks” section closes with concluding remarks and the study’s policy implications.

## Literature review

An extensive body of STIRPAT studies examine anthropogenic impacts on the environment. With regard to climate change, probably the most frequently studied issue in the STIRPAT environment, most studies confirm the role of a growing population and increasing affluence on CO_2_ emissions (e.g., Kenworthy and Laube 1999; Lankao et al. 2009; Karathodorou et al. 2010; Liddle and Lung 2010; Travisi et al. 2010; Xu and Lin 2016; Ge et al. 2018; Lv et al. 2019; Amin and as well as Scholl et al. 1996 for OECD countries; Timilsina and Shrestha 2009 for Asian countries; Andrés and Padilla 2017 for the EU; and Dogan 2021 for regional studies).^2^

Compared to the rich portfolio of empirical studies related to greenhouse gases, the number of studies that analyze (local) air pollution is small, particularly for NO_x_ emissions, which are in the focus of the present study. However, Yang et al. (2020) analyze the potential impacts on NO_x_ emissions of 30 Chinese provinces and highlight the role of income and energy supply, which they suggest makes increasing denitrification tariffs a promising tool for reducing NO_x_ emissions. Applying a spatial regression technique for Chinese provinces, Diao et al. (2018) confirm the significant and positive impacts of income (GDP per capita) on NO_x_ emissions for the period from 2006 to 2015. While they also identify significant impacts from population size, energy efficiency, and the industrial structure, their results indicate no significant impact from the number of private vehicles. This finding is in contrast to Montero et al. (2021), who analyze the drivers of NO_x_ emissions in communities in the Madrid area from 2000 to 2009 and find clear impacts of the number of vehicles. Their findings also point to spatial effects and a strong impact of affluence on NO_x_ emissions.

Most STIRPAT studies confirm the roles of a growing population and increasing affluence, typically measured by the number of inhabitants and GDP per capita, respectively, on the environment. The advantage of these measures lies in their simplicity, as well as availability of good data, which allows conclusive comparisons and policy implications at the macro level. For example, many empirical studies find that population has clearly higher ecological elasticity than economic growth, which some authors take as a reason to argue in favor of a slowed economy and reduced population growth (e.g., Casey and Galor 2017). Even though some authors are critical of the feasibility and effectiveness of population policies, the broad consensus is that population growth must be considered as having significant environmental impacts.

At the same time, causal relationships between population and environmental impacts are not as simple as they appear. For example, empirical findings at the regional and city level suggest that population’s environmental impacts do not necessarily relate to the number of residents so much as the age structure, household size, number of households, and education level (Cramer 1998; Liddle and Lung 2010; Liddle 2011; Zagheni 2011; York and Rosa 2012), because consumption patterns vary substantially for different age cohorts, stages of life, and education levels (Liddle 2013b). Some studies also pay attention to the EKC relationship between population and environmental outcomes and include a quadratic term of population. Although these studies’ results are so far inconclusive, Cole and Neumayer (2004) demonstrate that, in the case of SO_2_ emissions, a quadratic effect can be observed in some situations. This result suggests that the population-emissions elasticity is negative for small population sizes but rises rapidly as population increases.

In contrast to a differentiated understanding of population, most STIRPAT applications treat affluence as one dimensional. Although several authors emphasize the limitations of GDP per capita as a measure of affluence (e.g., Kashima and Kashima 2003; Majewska and Gierałtowska 2022) and underscore the importance of differentiating the role of affluence more fully, particularly by accounting for consumption and production effects (Ehrlich and Holdren 1971; Dietz and Rosa 1994, 1997; Waggoner and Ausubel 2002; York et al. 2003), empirical applications to date tend to stick to the easily available measure of GDP per capita.

Notable exceptions differentiate between GDP and public infrastructure per capita (Li et al. 2017), account for electric power consumption and sectoral value added (Montero et al. 2021), or use sectorally disaggregated GDP (Arshed et al. 2021; Wang et al. 2021). Some studies address the EKC relationship and include quadratic forms of (sectorally disaggregated) GDP per capita (e.g., Dietz and Rosa 1997; York et al. 2003; Arshed et al. 2021; Wang et al. 2021), while others expand the STIRPAT approach to the marketing industry and include elements of consumer behavior, such as consumer spending and consumption of material goods (Kilbourne and Thyroff 2020). However, the focus there is on the theoretical expansion of STIRPAT to the marketing industry and not on empirical application, as only a cross-country regression for 1 year is applied. Studies outside the STIRPAT literature that examine the environmental impacts of affluence also point to the role of housing conditions, mobility patterns, socioeconomic status, and income distribution (e.g., Dunlap and Mertig 1995, Myers and Kent 2003; Ransome 2005; Boyce et al. 2006; Peichl and Pestel 2013; Weinzettel et al. 2013; Hobza et al. 2017; Törmälehto 2017; Majewska and Gierałtowska 2022).

## Decomposition of affluence

In an attempt to provide a differentiated view of affluence’s impacts on the environment, we decompose affluence into three parts: taxable income per taxpayer (instead of the more common GDP per capita), car ownership (private passenger cars per capita), and the number of single-family houses per capita.

Because of rising profit shares in most OECD countries, in recent years, real GDP generally increased at a much faster pace than real household income did. However, the related literature indicates that household income, rather than GDP, is the basis of material wealth for most people and determines consumption patterns (Alda et al. 2004; Ribarsky et al. 2016). Therefore, we use taxable income per taxpayer instead of GDP per capita as a first measure of affluence.^3^

Second, affluence can also be measured by the level of personal car ownership in a region. This is because of the related cost of acquisition and maintenance (Galobardes et al. 2006; Lansley 2016). Even though some recent findings of increasing rates of ownership among the poor and a carless but affluent young generation in metropolitan areas indicate a decoupling of car ownership and social standing, car ownership still relates strongly to regional income levels in developing nations (e.g., Li et al. 2010 (for Chinese regions); Huang et al. 2012 (for Chinese cities)), as well as highly industrialized nations (e.g., Yeboah et al. 2007 (for England and Wales)).

Finally, the number of single-family houses per capita reflects not only a region’s settlement structure and housing situation. Due to higher construction and maintenance costs, a higher per-capita share of single-family houses further relates to a region’s level of affluence (Kohler et al. 2017).

Eventually, decomposing affluence into car ownership, share of single-family houses, and taxable income per taxpayer allows for a more differentiated analysis of environmental impacts. As the rate of car ownership substantially increases traffic density, it can be seen as a key driver of local air pollutants (Mayerthaler et al. 2017). Given the unbroken increase in private car ownership in Germany, we propose that this aspect of affluence substantially contributes to the production of NO_x_ emissions. Considering single-family houses, building characteristic (e.g., living space per person or smart home devices) as well as the occupants’ behavioral patterns (e.g., usage of home office, home entertainment systems, or private spa areas) can increase the per-capita energy consumption of single-family houses over that of other residential buildings (Yohanis et al. 2008). Therefore, the share of single-family houses per capita can be expected to correlate positively with local NO_x_ emissions. In contrast to the impacts of car ownership and the share of single-family houses, the impact of income seems not clear. On the one hand, empirical findings of most STIRPAT studies indicate that increasing income positively correlates with emissions. On the other hand, following the main EKC hypothesis, increasing income could come along with higher willingness to pay for environmental protection (see the “Literature review” section). This particularly holds for local pollution, where environmental spending transfers into noticeable improvements of the situation. Following this line of thought, we assume that taxable income relates negatively to the development of air pollutants such as NO_x_ emissions (if we control for the emission-intensive activities of affluence, such as car ownership and housing situation).

## Theoretical model and empirical application

### STIRPAT model

The STIRPAT approach was developed from the IPAT identity, which states that environmental impacts (*I*) are the multiplicative products of population (*P*), affluence (*A*), and technology (*T*) (Commoner et al. 1971; Ehrlich and Holdren 1971). That is,

1 $$I=P\bullet A\bullet T.$$

While its clarity and simplicity add to the popularity of the IPAT approach, the pure identity undermines hypothesis testing and causal interpretation (e.g., York et al. 2003). Therefore, Dietz and Rosa (1994) suggest transferring the IPAT equation into the STIRPAT model, which explains stochastic impacts on the environment by regression on population, affluence, and technology and provides the framework for empirical analysis:

2 $${I}_{i,t} ={c}_{t}\bullet {P}_{i,t}^{\alpha } \bullet {A}_{i,t}^{\beta } \bullet {T}_{i,t}^{\gamma } \bullet {e}_{i,t},$$

where *I*_*i*,*t*_ is the environmental impact of country *i* at time *t*, *P*_*i*,*t*_ is population, *A*_*i*,*t*_ is affluence, *T*_*i*,*t*_ is technology, ${c}_{t}$ is the constant, and *e*_*i*,*t*_ is the residual error term. *α*, *β*, and *γ* are the economic outcome elasticities with respect to population, affluence, and technology, respectively.

After taking the logarithm, the model is set up according Eq. (3):

3 $$\ln\;I_{i,t}=\ln\;c_t+\alpha\bullet\ln\;P_{i,t}+\beta\bullet\ln\;A_{i,t}+\gamma\bullet\ln\;T_{i,t}+\ln\;e_{i,t}.$$

The logarithmic form of the STIRPAT equation provides a tractable regression equation and dampens the potential for a skewed distribution of the variables (Jorgenson and Clark 2010).

In decomposing affluence into the three dimensions, we estimate Eq. (3) by regressing NO_x_ emissions on population, taxable income per taxpayer, car ownership, and share of single-family houses per capita. Given the significant role of industrial emissions, we control for the share of industrial manufacturing and assume a positive impact. Finally, and in line with most regional studies, the model includes urban density, as we assume the well-established negative relationship between urban density and CO_2_ emissions (Kenworthy and Laube 1999; Lankao et al. 2009; Karathodorou et al. 2010; Travisi et al. 2010; Liddle 2013b) because of urban areas’ more efficient energy use by the housing sector and more favorable conditions for public and non-motorized individual transport.^4^

### Data

We used a balanced cross-regional panel dataset (1990–2020) of 367 German districts and autonomous cities for the empirical application (NUTS 3).

The German Environment Agency (Umweltbundesamt 2021) provides data on regional emissions in the form of total NO_x_ emissions measured in kilotons. Although (local) concentrations of nitrogen oxides have generally declined over time, they still exceed policy targets and have been associated with serious impacts on health (e.g., asthma, hypertension, diabetes mellitus) in both rural districts and autonomous cities (Schneider et al. 2018). The data are available for a 5-year interval.

Statistics from the Statistical Offices of the Federation and Lands (Statistische Ämter des Bundes und der Länder 2021) identify increasing income per taxpayer (measured in €) for the 1990–2020 period we considered, albeit with regional differences. Population, which largely varies with the regions’ sizes and urbanization levels, is generally increasing in the cities but stagnating or even shrinking in rural districts (Statistisches Landesamt Baden-Württemberg 2021).

Data from the Federal Motor Vehicle Office (Kraftfahrt-Bundesamt 2022) shows that the average rate of private car ownership in Germany continuously increased from an already high level of just below 500 cars per 1000 inhabitants in 1990 to more than 550 cars per 1000 inhabitants in 2020 (+ 14%) with no likely future breaks in the trend. Although the national trend is driven by rural districts, where the average rate of car ownership increased by more than 25% between 1990 and 2020, from 491 to more than 618 cars per 1000 inhabitants, car ownership is also increasing in most German cities.

The number of single-family houses per capita has increased over time and averaged 18 per 1000 residences in 2020 (Statistische Ämter des Bundes und der Länder 2021).

Urban density can be defined in various ways in STIRPAT analyses (Dovey and Pafka 2014). We follow the most common measure, inhabitants per square kilometer. Of course, average urban density (279 inhabitants per km^2^) is much higher and increases faster in the cities than it does in other districts. However, these dynamics vary widely across regions. For example, districts that surround major cities have similar or even more dynamic trends than cities themselves, probably because of lower land prices, less congestion, and more possibilities for expansion (Statistisches Bundesamt 2021).

The State Office for Statistics Baden-Württemberg (Statistisches Landesamt Baden-Württemberg 2021) reports that industrial manufacturing was 34% of the GDP in 2020. The share of industrial manufacturing is a measure of the industrial structure of an economy (Cole and Neumayer 2004).

**Fig. 2:**
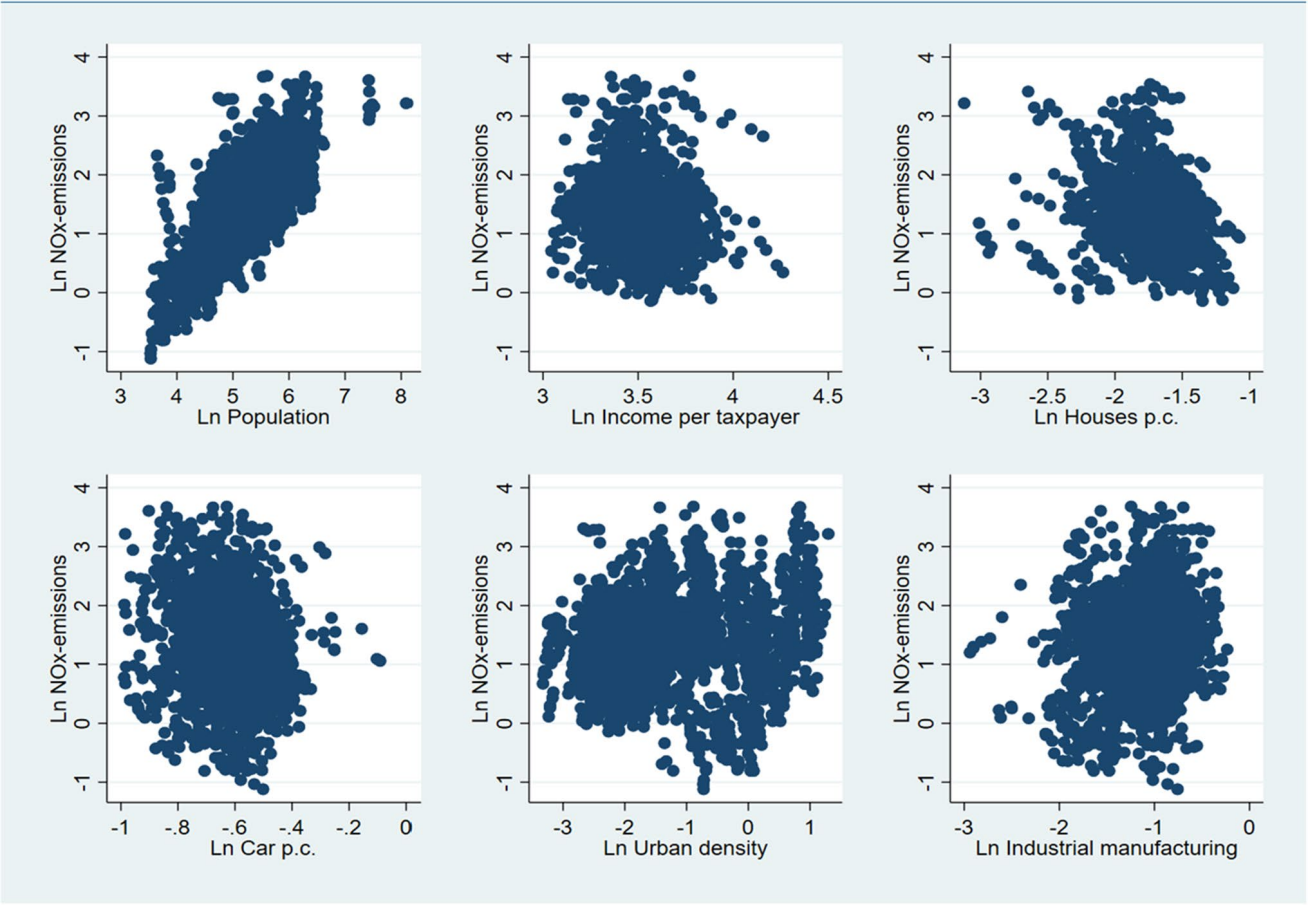
Scatterplots of NO_x_ emissions and the main explanatory variables

Table 1 summarizes definitions, means, standard deviations, minima, maxima, skewness, and kurtosis of the variables used in the study. The scatterplots in Fig. 2 indicate the correlations between NO_x_ emissions and the main explanatory variables.

**Table 1:** Definitions and statistical descriptions of the study’s main variables

Variables	Definition	Mean	Standard deviation	Minimum	Maximum	Skewness	Kurtosis
NO_x_ emissions	Kilotons	5.13	4.69	0.33	39.66	3.06	15.93
Population	Thousand	198.53	223.88	34.14	3629.16	9.59	128.93
Income per taxpayer	Income (€)/taxpayer	32803.55	6587.33	17172.94	70936.16	0.93	5.55
Car ownership	Cars/capita	0.55	0.07	0.21	1.14	0.45	9.29
Houses per capita	Houses/capita	0.18	0.05	0.04	0.34	0.03	3.26
Industrial manufacturing	% of GDP	34.13	11.08	5.29	79.09	0.38	3.52
Urban density	Inhabitants/km^2^	279.43	646.47	35.95	4072.58	2.35	8.52

### Model application

Application of the model starts with unit root tests to determine the (non-)stationarity of variables, so we applied the Im-Pesaran-Shin (IPS) test (with the null hypothesis that panels are *not* stationary). The results show that the variables’ levels (order of differences: 0) are stationary, allowing the null hypothesis to be rejected at the 0.01 significance level for all variables (Table 2).

**Table 2:** Panel unit root test

IPS test Order of differences: 0 *H*_0_: panels contain unit roots							
	NO_x_ emissions	Population	Income per taxpayer	Car ownership	Houses per capita	Industrial manufacturing	Urban density
*Z*-t-tilde-bar-statistic	− 38.77***	− 33.56***	− 17.70***	− 30.12***	− 18.27***	− 33.07***	− 31.47***

Im-Pesaran-Shin test (IPS test) assumes panel-specific AR parameters, Akaike Information Criterion is minimized, and all variables are logarithmized.

***p < 0.01.

We also tested the variables for panel cointegration. When variables are not cointegrated, the long-term relationship is only weakly defined and the short-term relationship can be calculated by estimating a first-differences equation. However, when variables are cointegrated, estimating first differences would ignore a potential long-term relationship of the key variables, so an error correction model should be applied to account for these dynamics (Engle and Granger 1987; Liddle 2011).

We applied the Kao and the Pedroni tests to check for cointegration (Table 3). All test statistics clearly reject the null hypothesis, which assumes no cointegration. Thus, strong evidence suggests a long-run cointegrating relationship among the variables, and we proceed by estimating long-run impacts using an error correction model.

**Table 3:** Results of the Kao and Pedroni cointegration tests

Kao test *H*_0_: no cointegration		Pedroni test *H*_0_: no cointegration	
NO_x_ emissions, population, income per taxpayer, car ownership, houses per capita, industrial manufacturing, urban density (all variables logged)			
Modified Dickey-Fuller *t* Dickey-Fuller *t* Augmented Dickey-Fuller *t*	− 19.58*** − 12.61*** − 7.14***	Modified Phillips-Perron *t* Phillips-Perron *t* Augmented Dickey-Fuller *t*	− 19.39*** − 18.70*** − 12.39***

The Kao test assumes a constant cointegration vector, and the Pedroni-test assumes panel-specific AR parameters. Cross-sectional averages are substracted

***p < 0.01

We used the fully modified ordinary least squares (FMOLS) estimator to estimate long-run elasticities. The FMOLS estimator can be applied to cointegrated panel data, and it addresses the cross-correlation between the cointegration equation error and the regressor innovations. The FMOLS estimator also accounts for any remaining non-stationarity issues and provides consistent estimates in small samples (Pedroni 2001; Chakraborty and Ghosh 2011). All variables are mean centered to mitigate potential structural multicollinearity problems and to get stable estimates (Raudenbush 1989; Cohen et al 2002; Bell and Jones 2015). The model is set up as in Eq. (3).

Table 4 presents the regression results with NO_x_ emissions as the dependent variable. In the first model setup, only population, taxable income per taxpayer, and car ownership are estimated. Then, single-family houses per capita, industrial manufacturing, and urban density are stepwise included. In addition, the model is estimated with GDP per capita instead of income per taxpayer.

**Table 4:** Determinants of NO_x_ emissions

Ln NO_x_ emissions	(1)	(2)	(3)	(4)	(5)	(6)
Ln population	0.90*** (0.05)	0.96*** (0.04)	1.02*** (0.06)	0.91*** (0.03)	0.95*** (0.03)	1.02*** (0.06)
Ln income per taxpayer (Columns (1)–(3)) Ln GDP per capita (Columns (4)–(6))	− 0.57*** (0.19)	− 0.41** (0.17)	− 0.49** (0.22)	− 0.15*** (0.20)	− 0.01 (0.22)	0.01 (0.11)
Ln cars ownership	0.77*** (0.25)	0.52** (0.23)	1.07*** (0.23)	0.98*** (0.06)	0.59* (0.08)	0.69* (0.37)
Ln houses per capita		0.32*** (0.09)	0.24** (0.12)		0.39*** (0.06)	0.28** (0.13)
Ln industrial Manufacturing			0.08 (0.08)			0.11 (0.08)
Ln urban density			− 0.04 (0.06)			− 0.09* (0.05)
Constant	0.072*** (0.08)	0.37*** (0.06)	0.36*** (0.07)	0.72*** (0.06)	0.41*** (0.07)	0.36*** (0.08)
***R***^2^	0.60	0.61	0.61	0.55	0.60	0.60
Number of districts	367	367	367	367	367	367
Number of observations	1315	1176	1167	2240	1168	1168

Robust standard errors are in parentheses. Year fixed effects are included. Variables are mean-centered before estimation, and estimations are based on the FMOLS technique. The varying number of observations is due to a lack of data for some variables

***p < 0.01; **p < 0.05; * *p* <0.1

In line with most STIRPAT analyses, population size positively and significantly affects NO_x_ emissions, a result that holds for all variations of estimation. For example, NO_x_ emissions rise by 0.90% when population rises by 1%.

The role of affluence is less conclusive. While private car ownership and the number of single-family houses per capita clearly translate into higher NO_x_ emissions for all estimations, the coefficients for taxable income per taxpayer are negative and significant in all cases. At first glance, the environmental impact of car ownership seems much greater than the impact of single-family houses per capita, but first estimations of standardized coefficients indicate no significantly different impacts of these variables (not shown). The results for population, private car ownership, and single-family houses per capita also hold if we replace taxable income per taxpayer with the more common measure of GDP per capita. However, the coefficients on GDP per capita are insignificant in two of three cases.

The coefficient for industrial manufacturing is positive but not significant. With regard to urban density, the coefficient behaves as expected in indicating a negative impact on emissions. However, the coefficient is only strongly significant when GDP per capita is used instead of income per taxpayer.

Next, we evaluate the quality of the data and results using postestimation statistics and variations of estimations. First, we control for multicollinearity, so we calculate the independent variables’ variance inflation factors (VIFs). The VIF indicates how much of the variance in the estimated regression coefficient would be inflated if the independent variables are correlated. The calculated values are clearly below 10 (maximum of 3.69), indicating that multicollinearity is unlikely to be a problem (Shrestha 2020; Table 5).

**Table 5:** Postestimation statistics

	Results based on regression model in Table 4 column (3)	Results based on regression model in Table 4 column (6)
	VIF	
Ln population	1.98	1.98
Ln income per taxpayer	3.17	
Ln GDP per capita		1.91
Ln car ownership	3.07	2.67
Ln houses per capita	2.16	2.22
Ln industrial manufacturing	1.13	1.10
Ln urban density	3.69	3.20
	Model specification (link test)	
Prediction squared	− 0.01 (not significant, *p* > 0.1 )	0.01 (not significant, *p* > 0.1)

Second, we test whether the regression equation is misspecified because of missing variables or the assumption of the functional form. We perform a link test by regressing the independent variable to its prediction and its prediction squared. The results show that the null hypothesis, according to which there is no specification error, cannot be rejected—that is, the prediction squared has no explanatory power (Table 5)—so there is no evidence of misspecification in the model (Alho and Silva 2014; StataCorp. 2017).^5^

Finally, we estimated the model for other time periods (e.g., 1995–2015 and 2000–2020; not shown). The results remain qualitatively and quantitatively similar, confirming the robustness of the coefficients.

## Discussion of results

Our findings show that the development of NO_x_ emissions is clearly related to population, car ownership, the housing situation, income per taxpayer, and urban density in German districts and autonomous cities. While private car ownership, the number of single-family houses per capita, and population positively affect NO_x_ emissions, taxable income per taxpayer and urban density have negative effects. Moreover, the significant results for the decomposed dimensions of affluence reveal a varying role of affluence in environmental degradation.

The positive impact of car ownership on NO_x_ emissions reflects an increase in motorized passenger transport in almost all counties and cities. Given the high share of cars with traditional combustion engines, which is particularly pronounced in rural districts but is also observed in most of the cities, individual motorized transport will remain a driver in local pollution in the near future. However, the emergence of e-mobility could change the game in the medium and long runs. In that case, even if private car ownership continues to increase, local emissions related to the internal combustion of fossil fuels may lose importance while other emissions (e.g., tire abrasion, brake dust) continue. For the moment, however, electric cars still account for less than 10% of new passenger car registrations.

The positive environmental impact of the housing situation is likely to relate to the comparatively high energy use per capita in single-family houses, particularly because of over-average heating consumption, which is still powered primarily by fossil fuels. However, other household-related consumption of electricity in smart homes, digital devices, and household appliances also contribute.

The negative correlation between taxable income per taxpayer and local NO_x_ emissions (when controlling for car ownership and the housing situation) could be explained by the higher educational attainment and the willingness to pay for an intact environment by those with higher income. Therefore, contrary to the common findings of the STIRPAT literature (which usually uses only GDP per capita to measure affluence), our results indicate a varying role of affluence on local emissions. This result may be due to the three dimensions of affluence capturing different aspects of wealth. While private car ownership and single-family houses could reflect the material- and energy-intensive part of affluence, taxable income per taxpayer covers (if we control for car ownership and the housing situation) expenditures for material (e.g., food, consumables) as well as types of consumption more common among the financially affluent (e.g., services, cultural activities).

The divergent impacts of the dimensions of affluence on emissions are in line with a limited number of STIRPAT studies that investigate affluence in a differentiated way (see the “Literature review” section). So, Montero et al. (2021) find a negative impact of gross disposable income and a positive impact of electric power consumption and sectoral value added on emissions (all of which indicate affluence) by analyzing the municipalities of Madrid. Furthermore, Arshed et al. (2021) show a U-shaped EKC for 80 countries when affluence is disaggregated into the sectorial shares of GDP (i.e., the industrial, agricultural, and services sectors). In contrast, Kilbourne and Thyroff (2020) find no qualitative differences in the environmental impacts of components of affluence like consumer spending and consumption of material goods in 113 countries.

In line with the classic STIRPAT analysis, our regional findings confirm the important role of population with respect to local NO_x_ emissions. In the STIRPAT literature, this effect is explained by the (high) level of energy consumption related to human activities.

Furthermore, our findings confirm the negative correlation between urban density and local pollution (NO_x_ emissions) that most empirical studies in this field find. More densely populated regions are likely to allow for more competitive public transportation and, because of shorter distances between probable destinations, more non-motorized individual transport.

Largely because of a lack of data at the district level, our analysis does not address some explanatory variables. While public transport structures and related activities might be captured, at least in part, by urban density (even though the quality of services differs among regions with similar density), weather conditions remain unconsidered. For example, the wind conditions mentioned above can have a significant impact on the concentration of local emissions (van Pinxteren et al. 2020).

Overall, the results presented here are robust to variations in the estimations used and confirm the appropriateness of the STIRPAT approach for estimating impacts on the local environment in small-structured regional settings (i.e., NUTS 3).

## Concluding remarks

The paper presents a region-based STIRPAT analysis that investigates anthropogeneous impacts on local air pollutants (NO_x_ emissions). Unlike most other regional studies, the analysis is not limited to a few cities but covers almost all German districts between 1990 and 2020. The paper decomposes affluence (one of the driving forces often identified) into three dimensions. Private car ownership, single-family houses per capita, and taxable income per taxpayer facilitate a more differentiated consideration of affluence and its environmental impacts.

Because of existing cointegration dynamics between variables, our findings are based on long-run estimation techniques and largely confirm the findings of related empirical studies (e.g., on the role of population and urban density). However, they also provide new evidence of major driving forces of NO_x_ emissions from a regional perspective. In particular, we find a varying effect of three dimensions of affluence on NO_x_ emissions, as private car ownership and single-family houses per capita can be considered drivers of local pollutants, but such is not the case for taxable income per taxpayer or GDP per capita (if the income variable is controlled for the other two dimensions of affluence).

Although our results are not generalizable outside their underlying regional sample, the analysis highlights the crucial roles of private car ownership and settlement structures in decisions regarding policies for fighting local air pollution and leads to three conclusions:

- Urban policies should further strengthen integrated mobility concepts with high shares of intermodal transport, easily accessible car-sharing services, and so on. Mobility patterns can be highly persistent and, because of socio-demographic or topographic conditions, highly dependent on private cars, not only particularly for rural regions but also for smaller cities. Therefore, the call for better public services and more bike lanes could fall short of the mark, so they should be complemented with policies that support the transition to low-emission car technology.
- Policies should further support low-emission infrastructure (e.g., local and district heating networks) to mitigate its environmental impacts that are due to existing housing conditions and related consumption patterns. In addition, incentives should be established that favor investment into modern heating and self-sufficiency systems (e.g., insulation, photovoltaic installations, energy efficient appliances).
- Considering a more general aspect of STIRPAT modelling, our findings encourage a differentiated view of the role of affluence (or economic growth) in environmental degradation. While some dimensions of affluence can be considered drivers of emissions (e.g., private car ownership and single-family houses), other dimensions of affluence might work in the other direction (e.g., taxable income). Hence, future research is needed to understand fully the various impacts of affluence on the environment.

Like most empirical studies, the analysis could benefit from additional control variables that facilitate a more in-depth analysis of anthropogenic drivers of environment degradation. For example, detailed information on local freight transportation, which can be considered an important source of NO_x_ emissions, could be of value, as could knowing more about the age structure of single-family houses or the fuels used for heating. However, data, particularly time-series data, at the regional level is limited. Future analyses could focus on specific regions with better data availability (e.g., cities) to examine these factors. With regard to the rapid shift to electric cars and the mandatory installation of photovoltaic systems on new houses (at least in some regions), adopting a 1-year interval and predicting future trends could be useful.

## Data Availability

The data are available on request.

## Footnotes

1 In fact, Germany’s average concentration of NO_x_ is among the highest in the EU, and almost all registered values are above the WHO guideline.

2 While cross-country comparisons typically control for trade and economic structure or complexity, regional and city-based studies generally account for population (or urban) density and transport-related issues.

3 However, to allow a better comparison with other studies, we also run the model with GDP per capita (see the “Model application” section).

4 Aside from STIRPAT modelling, some studies control for climate functions and meteorological conditions that could, favorably or not, affect NO_x_ concentrations. For example, the findings of a recent study by the Leipniz Institute for Tropospheric Research (van Pinxteren et al., 2020) indicate that wind speed relates negatively and significantly to NO_x_ concentrations, so wind-protected regions in *bowl* or *basin* locations have higher concentrations. Empirical findings on other meteorological factors (e.g., temperature, precipitation, and solar radiation) are not yet conclusive, although these factors seem to have small or no impacts. These conditional factors remain largely unconsidered in STIRPAT models, which have a clear focus on anthropogenic drivers.

5 Similarly, we test for potential non-linear relationships between NO_x_ emissions and income per taxpayer and between NO_x_ emissions and population. Overall, we did not find evidence for the inclusion of squared terms, as only when population, population squared, and income are used as explanatory variables does a significant negative impact of population squared appear (not shown). This result might be due to our use of disaggregated variables for affluence and control variables’ (for technology) catching-up potential non-linearities (Cole and Neumayer, 2004). Furthermore, the necessary threshold level for EKC is likely to be out of the sample range used here.
